# Vici Syndrome: A Rare Autosomal Recessive Syndrome with Brain Anomalies, Cardiomyopathy, and Severe Intellectual Disability

**DOI:** 10.1155/2011/421582

**Published:** 2011-06-22

**Authors:** R. Curtis Rogers, Bridgette Aufmuth, Stephanie Monesson

**Affiliations:** Greenwood Genetic Center, Greenville Office, 14 Edgewood Drive, Greenville, SC 29605, USA

## Abstract

*Purpose*. The objective of this study was to present and describe two additional patients diagnosed with Vici syndrome. *Methods*. Clinical, laboratory, and imaging findings of the two siblings are discussed in detail. The two patients' descriptions are compared with the other eleven patients reported in the literature. We also presented detailed autopsy results on the male sibling, which demonstrated cytoplasmic vacuoles of the cardiomyocytes and confirmed the clinical findings. *Results*. The patients reported here include the 13th and 14th patients reported with Vici syndrome. The summary of findings present in these patients includes postnatal growth retardation, developmental delay, bilateral cataracts, agenesis of the corpus callosum, cerebellar anomalies, gyral abnormalities, seizures, hypotonia, and cardiomyopathy. *Conclusion*. Vici syndrome should be suspected in any child with agenesis of the corpus callosum and one of the following findings: cardiomyopathy, cataracts, immune deficiency, or cutaneous hypopigmentation.

## 1. Introduction

Vici syndrome was first described in 1988, in two brothers with agenesis of the corpus callosum (ACC), bilateral cataracts, cleft lip and palate, hypopigmentation of the skin and hair, combined immunodeficiency, and severe psychomotor retardation [1]. The patients died at ages 2 and 3 years from bronchopneumonia. Two other siblings and two additional patients were described by del Campo et al. [2]. The major clinical features shared by all patients were ACC, recurrent infections, hypopigmentation of the skin and hair, hypotonia, poor postnatal growth, and developmental delay. The two siblings, a brother and a sister, died at age 2 years and age 11 months due to myocardial failure and apnea, respectively. One male patient died at age 16 months following progressive deterioration of cardiac function, and the final patient, a female, was still living at age 3.5 years. Based on the presence of the clinical features in two siblings in two separate families, the pattern of autosomal recessive inheritance was established. 

A third report in the literature by Chiyonobu et al. [3] presented two additional siblings, a brother and a sister, with Vici syndrome. The sister died at age 19 months, possibly due to progressive myocardial failure, while the brother was still living at age 6 months. Miyata et al. [4] reported sibling cases of Vici syndrome with complications of renal tubular acidosis. The sister died at 12 months due to heart failure, and the brother was still alive at 13 months. Another report of a patient with Vici syndrome was given by McClelland et al. [5]. This patient presented with hypotonia, feeding difficulties, hypertrophic cardiomyopathy, atypical facies, bilateral cataracts, recurrent infections, and profound bilateral sensorineural hearing loss. The patient died at 3 months due to deterioration of cardiac function.

An additional report of Vici syndrome was recently published by Al-Owain et al. [6]. The male had three deceased siblings (2 boys, 1 girl), all with clinical findings reminiscent of Vici syndrome. The patient died at 9 months as a result of sepsis, and an autopsy was not performed. He was the longest living of his affected siblings. Electromyography (EMG) revealed a myopathic pattern and pseudomyotonic discharges, and a muscle biopsy showed marked variation in myofiber size, scattered atrophic fibers, and rare degenerative and regenerative fibers. The Gomori trichrome stain revealed a subsarcolemmal vacuolar change and a focal mild increase in endomysial connective tissue. The Periodic Acid-Schiff (PAS) stain indicated a buildup of glycogen in many vacuoles. Nonspecific muscle biopsy findings were also reported in the patients of del Campo et al. [2] and McClelland et al. [5]. 

In this paper we introduce two new patients diagnosed with Vici syndrome, a brother and a sister who both died at age 8 years. The autopsy findings of the male sibling are reviewed, and the neurological findings in the reported patients are summarized.

## 2. Materials and Methods

### 2.1. Patients

#### 2.1.1. Patient 1

**Figure 1:**
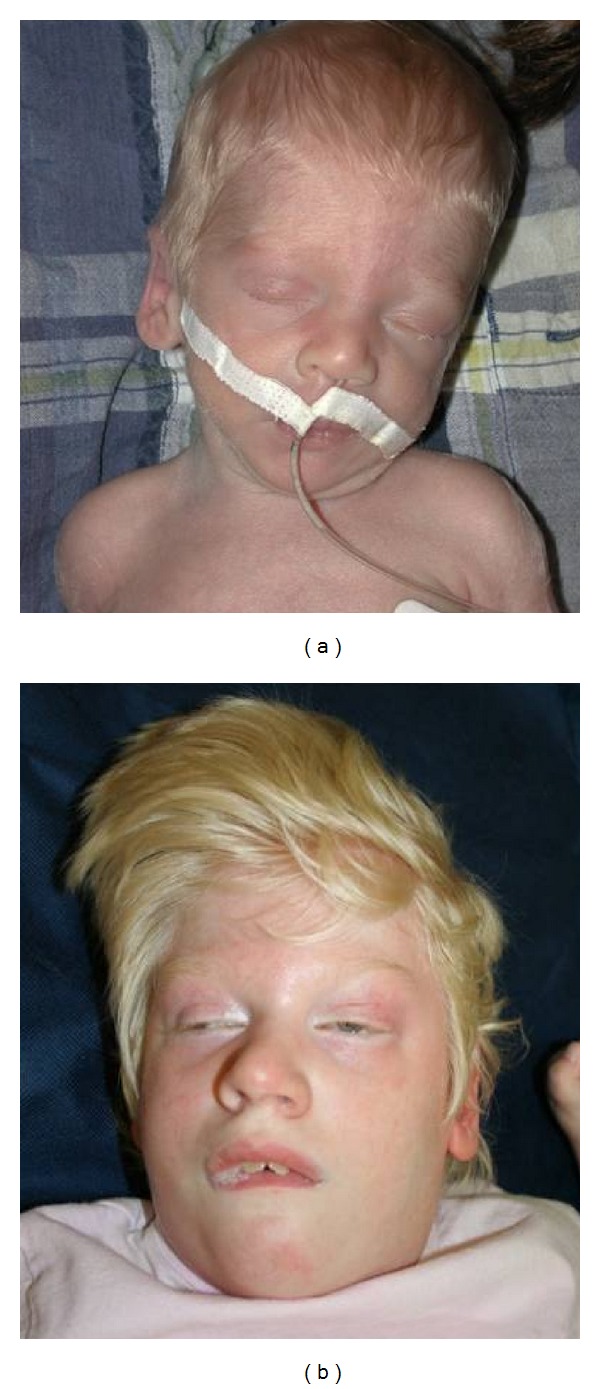
(a) Patient 1 and his sibling, (b) Patient 2, at 2 weeks and 6 years, respectively. Both patients have hypopigmentation and profound intellectual disability.

Patient 1 was the first pregnancy for his parents, a nonconsanguineous couple. He was born at 38 weeks gestation after an uncomplicated pregnancy. His birth weight was 2.46 kg, length was 48.3 cm, and head circumference was 32.5 cm. At age 11 days, he experienced an episode of respiratory failure and cardiac arrest. Imaging studies revealed ACC, enlarged ventricles, hypoplasia of the cerebellar vermis and brain stem, and cardiomyopathy with biventricular hypertrophy. Physical examination revealed mild nystagmus, a high arched palate, fair complexion, overlapping toes, and hypotonia (Figure 1(a)). Plasma amino acids, urine amino acids, urine organic acids, serum transferrin isoelectric focusing, and urine metabolic screen were normal. Chromosome studies revealed a 46XY karyotype. Immunological studies showed decreased absolute T cells, T helpers, and T suppressors, indicating T-cell-mediated immunodeficiency. Bilateral cataracts were identified at age 4 months. The patient also developed hypothyroidism and had a history of both seizures and recurrent infections, including pneumonia and urinary tract infections.

 The patient was lost to follow up until his sister was diagnosed prenatally with ACC during a fetal ultrasound. At age 6 years, the patient was reported by his parents to have improvements in seizure frequency and less severe infections but had significant developmental delay. No significant copy number variants were noted using the Affymetrix Genome-Wide SNP 6.0 Microarray system. Several small blocks of homozygosity (LCSH)/loss of heterozygosity (LOH) regions were observed (>86 Mb), encompassing greater than 3.01% of the individual's genome. The largest block for this patient was on Chr3: 95741944-98847764 (3.1 Mb) which is below the threshold of clinical significance. 

Patient 1 died at age 8 years due to progressively decreasing cardiac function. The autopsy findings included agenesis of the corpus callosum and cerebellar vermis, absent thyroid and hypoplastic appearing thymus, and a profoundly enlarged heart with biventricular dilation. Hypopigmentation of the retina and fovea was also present. Left ventricular sections depicted regions of hypertrophied and vacuolated cardiomyocytes, as well as interstitial and replacement fibrosis. Similarly, right ventricular sections demonstrated hypertrophied and vacuolated cardiomyocytes, but to a much lesser extent, and did not contain interstitial or replacement fibrosis. The enlarged and vacuolated cardiomyocytes contained large, irregular nuclei and membrane-bound cytoplasmic inclusions within the cardiomyocytes containing granular material with a “ribbon-like” appearance. The intracellular inclusions stained positively with Periodic Acid-Schiff (PAS), and staining was resistant to diastase digestion. Focal areas of scarring due to ischemic necrosis were seen in both kidneys. Lung tissue sections were consistent with an organizing pneumonia, and multifocal areas demonstrated chronic inflammation with fibrosis and multinucleated giant cells. Some sections of the cerebellum showed a mild increase in foveal space with appropriate granular layer and minimal Purkinje cell loss, while others showed a high increase in intrafoveal space with degeneration or loss of granular cell layer and significant Purkinje cell agenesis. The leptomeninges were thin and delicate. Mild polymicrogyria was present in the occipital regions of the cerebral hemispheres. Within the deep tissue structures of the brain, specifically the basal ganglia, there was poor gray/white matter demarcation. Other brain abnormalities included moderate dilation of the third ventricle (1 cm) and duplication of the septum pellucidum. No significant histopathological abnormalities were seen in the skeletal muscle, lymph nodes, skin, or adrenal glands.

#### 2.1.2. Patient 2

Patient 2, the sister of Patient 1, was the third pregnancy for her parents. Prenatal ultrasound showed intrauterine growth retardation, oligohydramnios, and agenesis or hypoplasia of the corpus callosum. The child was delivered by c-section at 33 weeks gestation for late decelerations and oligohydramnios. The patient was premature and remained in the NICU for the first six weeks of life. Her birth weight was 1.225 kg, length 40 cm, and head circumference 26.5 cm. Initial evaluation revealed hypotonia, ACC, cardiomyopathy, mild micrognathia, and cutaneous syndactyly of toes 2 and 3 (bilaterally). By age 2 months, she had developed bilateral cataracts, and her height, weight, and head circumference were less than the 5th percentile. She had also developed a seizure disorder and mild cardiomegaly. She and her father have a paracentric inversion of chromosome 2 [46,XX,inv(2)(q21.1q23)], not related to the disorder. Immunological studies showed normal absolute lymphocytes, absolute T cells, T helpers, and CD+3 absolute counts, and elevated CD+4 absolute counts. In addition to ACC, an MRI at age 2 years revealed prominent lateral ventricles, dysplasia of the cingulate gyrus, diffuse white matter atrophy, and atrophy of the brain stem and the proximal cervical cord.

 She was seen again at age 8 years and was undergrown with a weight of 20 kg, height of 117 cm, and a head circumference of 42.5 cm (5th–10th centile, 5th centile, and significantly less than the 5th centile, resp.). She displayed severe developmental delay. She also had spastic quadriplegia and severe scoliosis. Her hair, irides, and skin were pale. She had a narrow face with sharp features, a narrow, high palate with dental crowding, and an asymmetric chest wall (Figure 1(b)). Her pupils were unreactive to light, and the optic nerves were atrophic. She had extremely limited movement of her legs with contractures of her knees and ankles and milder contractures of the elbows. She had idiopathic dilated cardiomyopathy and a left ventricular shortening fraction of 34%. She has roughly 20 infantile spasms daily. Follow-up cardiac evaluations have demonstrated persistently borderline left ventricular function. The patient's respiratory status progressively worsened, and she died at age 8 years. An autopsy was not performed.

## 3. Results and Discussion

The siblings reported here represent the 13th and 14th patients clinically diagnosed with Vici syndrome. Both have postnatal growth retardation, developmental delay, bilateral cataracts, agenesis of the corpus callosum, cerebellar anomalies, gyral abnormalities, cardiomyopathy, seizures, and hypotonia.  Table 1 provides a summary of the clinical and imaging findings of the siblings that we describe and the other patients who have been reported in the literature.

Of the fourteen patients presented, nine are male and five are female. All reported patients (14/14) have agenesis of the corpus callosum, postnatal growth retardation, developmental delay, hypopigmentation of the hair and/or skin and/or retina, and hypotonia. Approximately 93% (13/14) of patients have atypical facies, cardiomyopathy, and recurrent infections/immune deficiency. Over half of the patients have cataracts (11/14), abnormalities of the palate (10/14), cerebellar abnormalities (9/13), seizures (9/14), and gyral abnormalities (8/13). Additionally, in the first patients reported by Vici et al. [1], one male was affected with hypospadias.

Table 2 shows a more comprehensive summary of the neurological abnormalities described in patients diagnosed with Vici syndrome. In addition to ACC, 6 patients reportedly had hypoplasia of the cerebellar vermis or cerebellar hemispheres, 5 patients had dilated ventricles, 4 patients had polymicrogyria/abnormal gyration, 3 patients had cerebral atrophy/hypoplasia, 2 patients had an absent septum pellucidum, and 2 patients had hypoplasia of the pons. In addition, there were single case reports of heterotopia and lung hypoplasia. Interestingly, the autopsy report of Patient 1 revealed a duplication of the septum pellucidum, a very rare finding (especially in conjunction with ACC).

While Vici syndrome is associated with many different congenital malformations, we suggest that this disorder should be suspected in any child with agenesis of the corpus callosum and one of the following findings: cardiomyopathy, cataracts, recurrent infections/immune deficiency, or cutaneous hypopigmentation. All the patients have severe developmental delay, and most have died within the first three years of life secondary to cardiomyopathy or pneumonia. Although our male patient did not show skeletal muscle abnormalities, the muscle biopsy findings in the patients of del Campo et al. [2], McClelland et al. [5], and Al-Owain et al. [6] indicate that Vici syndrome may be a glycogen storage disorder. Further investigation is warranted to determine whether glycogen accumulation is a primary or secondary sign of Vici syndrome.

##  Conflict of Interests

 The authors have no financial, academic, or personal conflict of interests.

## Figures and Tables

**Table 1:** Summary of clinical and imaging findings in patients with Vici syndrome.

	Previous reports	Patient 1	Patient 2	Total
Sex	8 males, 4 females	Male	Female	9 males, 5 females
Poor growth	12/12	+	+	14/14
Developmental delay	12/12	+	+	14/14
Atypical facies	12/12	—	+	13/14
Cataracts	9/12	+	+	11/14
Palatal abnormalities	8/12	+	+	10/14
Hypopigmentation	12/12	+	+	14/14
ACC	12/12	+	+	14/14
Seizures	7/12	+	+	9/14
Hypotonia	12/12	+	+	14/14
Cardiomyopathy	11/12	+	+	13/14
Recurrent infections/immune deficiency	11/12	+	+	13/14

+ Indicates presence of symptom.

— Indicates absence of symptom.

**Table 2:** MRI findings in patients with Vici syndrome.

		ACC	Hypoplasia of cerebellar vermis/hemispheres	Hypoplasia of pons	Cerebral atrophy/ hypoplasia	Absent septum pellucidum	Dilated ventricles	Heterotopia	Polymicrogyria/ abnormal gyration
Past cases	(1)	+ (?)							
	(2)	+	+				+	—	—
	(3)	+	+				+	+	
	(4)	+	+				+		
	(5)	+				+			
	(6)	+							+
	(7)	+			+				
	(8)	+							
	(9)	+			+				
	(10)	+			+				
	(11)	+	+	+		+			
	(12)	+	+	+			+		+

Present cases	(13)	+	+	—	—	—	+	—	+
	(14)	+	—	—	—	—	—	—	+

+ Positive finding.

— Negative finding was specifically indicated in the literature.

(?) Patient 1 was assumed to have ACC due to positive ACC findings in sibling and clinical evaluation.

Blank space indicates that nothing was mentioned about that particular area in the literature.
